# 2166. Real-world susceptibilities to newer antibiotics in Enterobacterales and *Pseudomonas aeruginosa* including carbapenem non-susceptible and multi-drug resistant isolates : A multicenter analysis

**DOI:** 10.1093/ofid/ofad500.1789

**Published:** 2023-11-27

**Authors:** Todd Riccobene, ChinEn Ai, Kalvin Yu, Sara Gregory, Brooke Kim, Dmitri Debabov, Vikas Gupta

**Affiliations:** AbbVie Inc, Madison, New Jersey; BD - Becton, Dickinson and Company, Atlanta, Georgia; Becton, Dickinson and Company (BD), Franklin Lakes, New Jersey; Becton, Dickinson and Company, Franklin Lakes, New Jersey; AbbVie, Madison, New Jersey; Abbvie, Irvine, California; Becton, Dickinson and Company (BD), Franklin Lakes, New Jersey

## Abstract

**Background:**

Infections caused by multi-drug resistant (MDR) Gram-negative pathogens are increasingly problematic in healthcare settings and are associated with worse clinical outcomes in critically ill patients. We conducted a multicenter evaluation of real-world susceptibilities to newer antibiotics.

**Methods:**

Adult patients (≥ 18 years old) with facility reported antibiotic susceptibility from 2018-2022 across 100 facilities in the BD Insights Research Database (Franklin Lakes, NJ) were evaluated for overall and carbapenem non-susceptible (Carb-NS) Enterobacterales **(**ENT) and *Pseudomonas aeruginosa* (PSA) across respiratory, blood, urine, intra-abdominal, skin/wound, and other sources.

**Figure:**
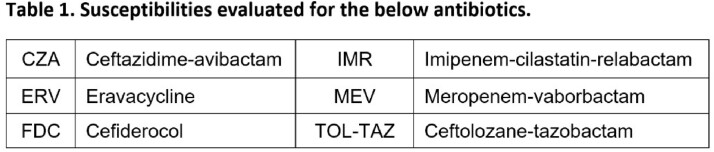

**Results:**

A total of 27676 susceptibility results for Gram-negative pathogens were available for one or more of the antibiotics in **Table 1** across 100 facilities, of which 79.9% (22101) and 19.6% (5420) were for ENT and PSA, respectively (**Table 2**). Ninety percent of susceptibility results for newer antibiotics were for *E coli* (42.9%), *P aeruginosa* (19.6%), *K pneumoniae* (14.6%), *P mirabilis* (8.1%), and *E cloacae* (4.8%). The top 3 antibiotics tested for ENT were CZA (11,667), TOL-TAZ (10,127), and MEV (211) and for PSA were TOL-TAZ (3172), CZA (1900), and FDC (317). For antibiotics reporting > 30 isolates, susceptibilities were highest for CZA in overall (98.7%), MDR (92.9%) and Carb-NS (85.0%) ENT and for FDC in overall (95.6%) and Carb-NS PSA (93.3%) (**Table 3**). Susceptibility of Carb-NS ENT to CZA was lower in 2022 (76.7%) than in 2018 (93.5%) but was consistent from 2019-2021 (85.7-87.7%). In Carb-NS ENT, TOZ-TAZ S was lower than CZA each year, ranging from 13.4 to 43.3% (**Figure 1**). CZA S was higher in 2022 than in 2018-2021 for Carb-NS PSA and was 82.0% compared to 86.4% for TOL-TAZ (**Figure 1**).

**Figure:**
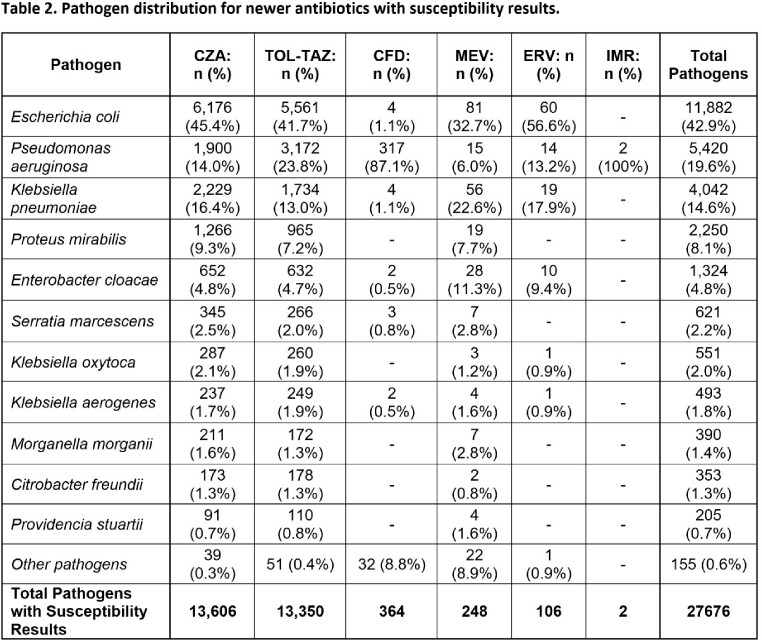

**Figure:**
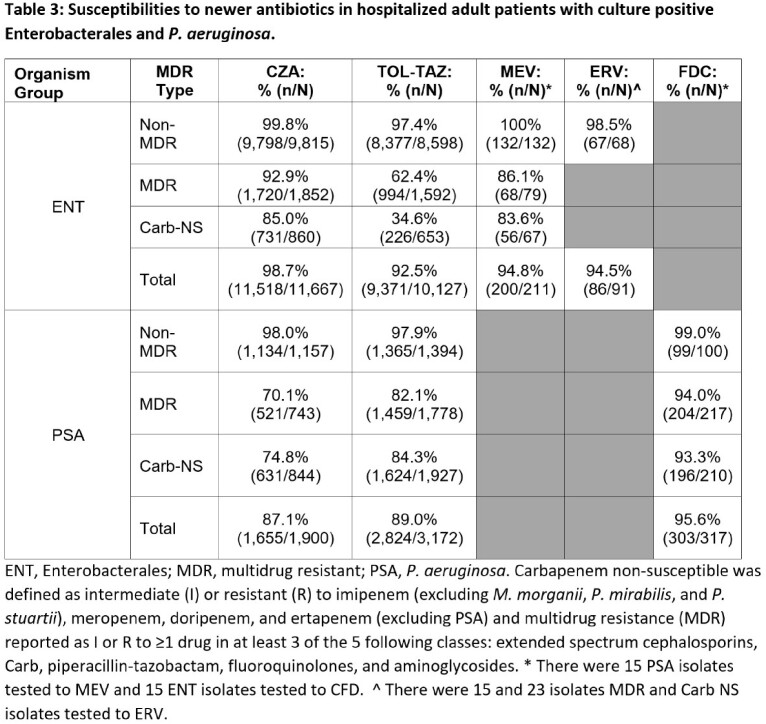

**Figure:**
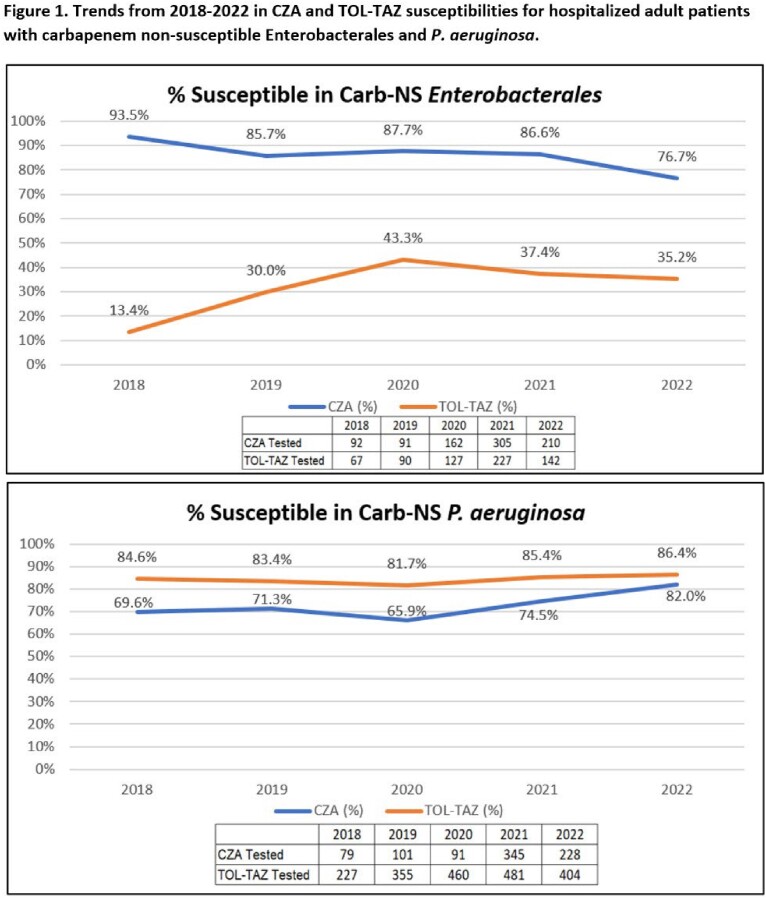

**Conclusion:**

Real-world susceptibilities of all ENT were > 90% to CZA, TOL-TAZ, and MEV. Susceptibilities of Carb-NS ENT to CZA and MEV were >80%. Real-world susceptibilities of all PSA were > 85% for CZA, TOL-TAZ, and FDC. Susceptibilities of Carb-NS PSA were > 70% to CZA, TOL-TAZ, and FDC. Newer antibiotics remain an important option for management of resistant ENT and PSA.

**Disclosures:**

**Todd Riccobene, PhD**, AbbVie: Employee salary|AbbVie: Stocks/Bonds **ChinEn Ai, MPH**, Becton, Dickinson and Company: Employee **Kalvin Yu, MD, FIDSA**, BD: Stocks/Bonds **Sara Gregory, PhD**, Becton, Dickinson and Company: Employee **Brooke Kim, RN, BSN, MSM**, AbbVie: Employee|AbbVie: Stocks/Bonds **Dmitri Debabov, PhD. Molecular biology**, Abbvie Inc.: Employee of Abbvie that participated in design, study conduct, financial support, interpretation of data, review, and approval of the publication **Vikas Gupta, PharmD**, Becton, Dickinson and Company (BD): Employee of BD|Becton, Dickinson and Company (BD): Stocks/Bonds

